# When rare meets routine: A case report of amyloid light-chain amyloidosis with multiorgan involvement in primary care

**DOI:** 10.51866/cr.1132

**Published:** 2026-09-13

**Authors:** Ilyana Sanusi, Mazapuspavina Md Yasin, Norashikin Saidon, Raja Ezman Raja Shariff, Nurfareha Mohd Hatta, Khairun Najihah Abd Khahar

**Affiliations:** 1 Department of Primary Care Medicine, Faculty of Medicine, Universiti Teknologi MARA, Sungai Buloh Campus, Sungai Buloh, Selangor, Malaysia.; 2 Department of Hematology, Faculty of Medicine, Universiti Teknologi MARA, Sungai Buloh Campus, Sungai Buloh, Selangor, Malaysia.; 3 Department of Cardiology, Faculty of Medicine, Universiti Teknologi MARA, Sungai Buloh Campus, Sungai Buloh, Selangor, Malaysia.; 4 Department of Pathology, Faculty of Medicine, Universiti Teknologi MARA, Sungai Buloh Campus, Sungai Buloh, Selangor, Malaysia.; 5 Department of Medicine, Faculty of Medicine, Universiti Teknologi MARA, Sungai Buloh Campus, Sungai Buloh, Selangor, Malaysia.; 6 Hospital Al-Sultan Abdullah, Universiti Teknologi MARA Puncak Alam, Malaysia.

**Keywords:** Systemic immunoglobulin light-chain amyloidosis, AL amyloidosis, Heart failure, Preserved ejection fraction, Proteinuria, Delayed diagnosis

## Abstract

Systemic immunoglobulin amyloid light-chain (AL) amyloidosis is a rare, life-threatening disease characterised by extracellular deposition of insoluble amyloid fibrils, leading to progressive multiorgan dysfunction. Its diverse clinical manifestations frequently overlap with common primary care conditions, delaying diagnosis. We report the case of a 70-year-old man with hypertension and coronary artery disease who presented to a primary care clinic with bilateral lower limb oedema and haematuria. By the 7th month, systemic AL amyloidosis was clinically suspected based on evolving multisystem findings. Serial investigations revealed unexplained significant proteinuria, concentric left ventricular hypertrophy, heart failure with preserved ejection fraction and poor tolerance to standard heart failure therapy. The new appearance of periorbital purpura and macroglossia, together with markedly elevated cardiac biomarkers and electrocardiographic—echocardiographic discordance, reflected features of restrictive cardiomyopathy, further raising suspicion for systemic AL amyloidosis. Renal biopsy demonstrated Congo red-positive amyloid with lambda light-chain restriction; bone marrow examination revealed clonal plasma cell proliferation; and serum free light-chain assay confirmed monoclonal lambda light chains, fulfilling the International Consensus Classification 2022 diagnostic criteria for systemic AL amyloidosis. Definitive diagnosis was established at the 9th month. The haematology team initiated anti-clonal therapy with bortezomib, cyclophosphamide and dexamethasone; the patient died of pneumonia 3 months later, after the third treatment cycle. This case highlights the diagnostic challenges of systemic AL amyloidosis in primary care and the importance of recognising clinical discordance, pathognomonic signs and evolving multisystem involvement. Early identification and timely multidisciplinary referral are critical to optimising outcomes in this rare but devastating disease.

## Introduction

Amyloid denotes abnormal fibrous, extracellular, proteinaceous deposits in organs and tissues. Amyloidosis is a multisystemic disorder caused by accumulation of misfolded amyloid fibrils that resist normal catabolic processes. The classification of amyloidosis follows updated guidelines from the International Society of Amyloidosis Nomenclature Committee 2024, which identifies 42 human amyloidogenic proteins. It is based on the precursor protein (amyloid light chain [AL], transthyretin [ATTR], amyloid A [AA], leucocyte chemotactic factor 2 [ALECT2] and other hereditary forms), aetiology (acquired, mutant or iatrogenic) and clinical distribution (systemic versus localised).^1^ Systemic amyloidosis is more common than localised forms and is life-threatening because it affects multiple organs. The annual incidence of systemic amyloidosis ranges from 5 to 13 new cases per million people, with AL amyloidosis being the most common systemic subtype at approximately 10 cases per million people yearly.^2^

Systemic immunoglobulin AL amyloidosis or interchangeably called AL amyloidosis is a plasma cell dyscrasia characterised by tissue deposition of misfolded immunoglobulin light chains, which poses significant diagnostic challenges.^3^ Its clinical manifestations are non-specific and frequently overlap with common primary care conditions such as hypertensive heart disease, diabetic kidney disease or accidental bruising.^2^ Nephrotic-range proteinuria may be attributed to diabetic nephropathy, while early cardiac involvement presenting as heart failure with preserved ejection fraction (HFpEF) and concentric left ventricular hypertrophy (LVH) is often misinterpreted as hypertensive heart disease.^4^ As the dysfunction is primarily diastolic, the systolic ejection fraction (EF) remains normal until advanced stages, a classic feature of HFpEF.^5^ Pathognomonic findings such as periorbital purpura and macroglossia, although highly specific, occur in fewer than 15% of patients.^2^ Family physicians play a pivotal role in early recognition of AL amyloidosis. Diagnostic delay limits treatment efficacy and carries substantial prognostic implications.^4^ This case report illustrates the diagnostic challenges of AL amyloidosis in primary care, highlighting salient clinical clues, systematic evaluation and the importance of timely multidisciplinary referral.

## Case presentation

### Patient information

A 70-year-old man with hypertension, dyslipidaemia and coronary artery disease had been under follow-up at a university hospital primary care clinic since 2017, after opting for conservative management in preference to coronary artery bypass grafting. He had maintained a New York Heart Association class I functional status with medical therapy, comprising aspirin 75 mg, atorvastatin 80 mg, irbesartan 300 mg and amlodipine 10 mg daily. During a routine visit in November 2024, he reported 1 month of bilateral lower limb oedema and one episode of frank haematuria 2 weeks earlier. He had no angina, orthopnoea, paroxysmal nocturnal dyspnoea, fever, dysuria, constitutional symptoms or history of traditional medicine use. There was no family history of cardiac, renal or haematological disease.

### Clinical findings

Physical examination revealed bilateral pitting oedema to the mid-shin, blood pressure of 144/71 mmHg, a regular pulse of 94 beats per minute (bpm) and oxygen saturation of 98% on room air. He was neither cachectic nor pale. Cardiovascular and respiratory examination findings were unremarkable, with no jugular venous distension, organomegaly, lymphadenopathy, petechiae or bruising. Digital rectal examination was performed in view of the haematuria, revealing a normal prostate. Initial investigations, including complete blood count, liver function tests and coagulation profile, yielded normal findings, and renal function was preserved. Urinalysis was negative for infection but revealed significant proteinuria and microscopic haematuria without casts. The N-terminal pro-B-type natriuretic peptide (NT-proBNP) level was elevated (826 pg/ mL), and chest radiography showed borderline cardiomegaly. A working diagnosis of congestive heart failure was made, and he was started on frusemide 40 mg and bisoprolol 1.25 mg daily. Outpatient echocardiography (ECHO) and renal ultrasound were arranged. He continued follow-up in primary care for optimisation of guideline-directed medical therapy (GDMT) for heart failure.

Over the following 4 months, his condition progressively deteriorated despite optimisation of GDMT and maintenance of normotensive blood pressure. Mid-shin oedema persisted, and he developed new bilateral basal lung crepitations despite dietary modification and adherence to treatment. Other systemic examination findings were unremarkable. Serial investigations revealed declining renal function (estimated glomerular filtration rate [eGFR]: 65 mL/min/1.73 m^2^), progressive hypoalbuminaemia (32 g/L) and nephrotic-range proteinuria (3.03 g/24 h). Renal ultrasound showed no structural abnormality. ECHO demonstrated concentric LVH, grade 2 diastolic dysfunction and left atrial dilatation, with a preserved EF of 56% and a global longitudinal strain (GLS) of –18.2%. There were no regional wall motion abnormalities. At this point, diagnoses of HFpEF and nephrotic syndrome were made. He was referred to cardiology and started on spironolactone 25 mg and empagliflozin 12.5 mg daily, in addition to his existing GDMT. A nephrology referral was made, and renal biopsy was scheduled.

At the 7th-month follow-up, he reported worsening pedal oedema with giddiness despite normotensive blood pressure (123/71 mmHg) and regular pulse (86 bpm). Examination revealed periorbital purpura (Figure 1) and macroglossia (Figure 2A and 2B). Bilateral pitting oedema persisted below the knee. Laboratory investigations showed worsening hypoalbuminaemia, while renal function remained stable (eGFR: 67 mL/min/1.73 m^2^). Cardiac biomarkers were markedly elevated: high-sensitivity troponin T (hs-TnT) level of 145 ng/L and NT-proBNP level of 6373 pg/mL. Electrocardiogram (ECG) revealed low-voltage QRS complexes with pseudoinfarct Q waves in leads V2–V3 (Figure 3), discordant with the degree of LVH on ECHO. This constellation of evolving multisystem findings raised strong suspicion for AL amyloidosis, prompting urgent referral to a tertiary centre for multidisciplinary assessment.

**Figure 1:**
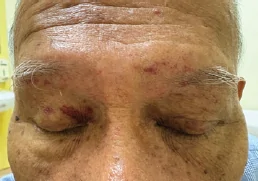
Periorbital purpura.

**Figure 2:**
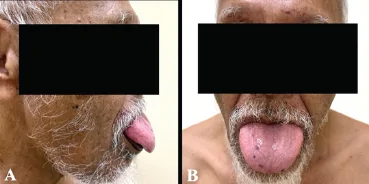
(A and B) Macroglossia with purpuric spots.

**Figure 3:**
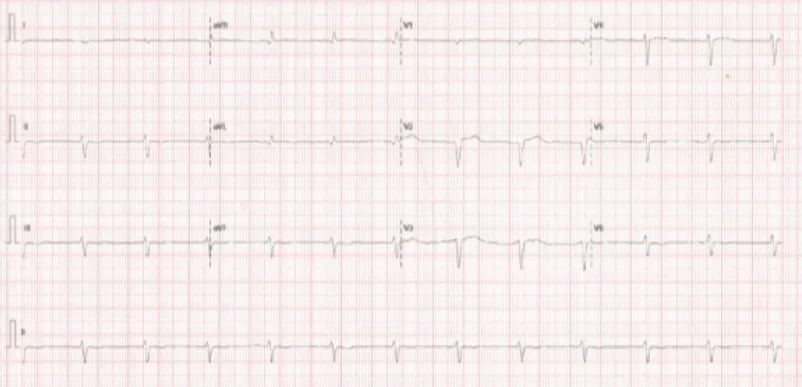
Low-voltage QRS complexes, discordant with left ventricular hyperplasia (S wave in V1 + R wave in V5 or V6 <15 mm) and pseudo-infarct pattern evidenced by Q waves in leads V2 and V3.

### Diagnostic assessment

Renal biopsy confirmed amyloid deposition with Congo red staining (Figures 4 and 5), and immunofluorescence demonstrated lambda light-chain restriction within the deposits. As mass spectrometry for definitive amyloid typing was unavailable in Malaysia, AL amyloidosis was inferred by integrating clinical, laboratory and biopsy findings and systematically excluding alternative diagnoses. Renal immunohistochemistry (IHC) for serum amyloid A (SAA) showed SAA-positive staining in the interstitium, vessel walls and perinephric adipose tissue, raising the possibility ofAA amyloidosis; however, this finding was interpreted with caution given the absence of clinical or laboratory evidence of chronic inflammatory, infectious or autoinflammatory disease and the recognised technical limitations of IHC-based typing.^6^ Serum free light-chain analysis demonstrated a markedly abnormal lambda-predominant ratio, and bone marrow examination confirmed a clonal lambda-restricted plasma cell population. Technetium-99m pyrophosphate scintigraphy showed no myocardial tracer uptake, effectively excluding ATTR amyloidosis.

Repeat ECHO revealed progressive biventricular hypertrophy (Figure 6), a reduced EF of 46% and a severely impaired GLS of -8.7% with a classic apical-sparing ‘cherry-on-top’ pattern (Figure 7). The serial investigations and diagnostic evolution are summarised in Tables 1 and 2. Overall, the integrated clinical, laboratory and histopathological findings supported a final diagnosis of systemic AL (lambda) amyloidosis with cardiac and renal involvement, fulfilling the International Consensus Classification (ICC) 2022 diagnostic criteria (criteria 1–4; Table 3). Cardiac biomarkers (hs-TnT level: 145 ng/L, NT-proBNP level: 6373 pg/mL) corresponded to European-modified Mayo 2004 stage Ilia disease.

**Figure 4:**
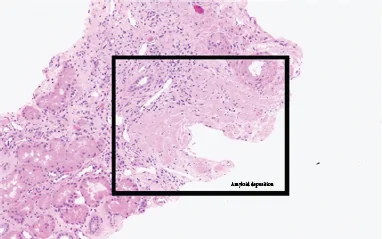
Histopathology of the renal biopsy sample (haematoxylin and eosin staining). Nodular mesangial expansion with thickened capillary loops and basement membrane spicules was observed, along with acellular, amorphous deposits in various structures.

**Figure 5:**
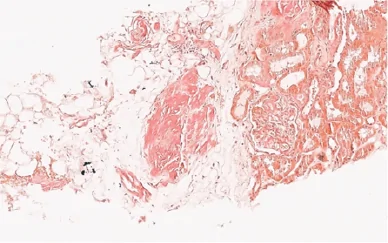
Histopathology of the renal biopsy sample (Congo red staining). Positive amyloid staining was observed in the mesangium, capillary loops, interstitium, vessel walls and perinephric adipose tissue.

**Figure 6:**
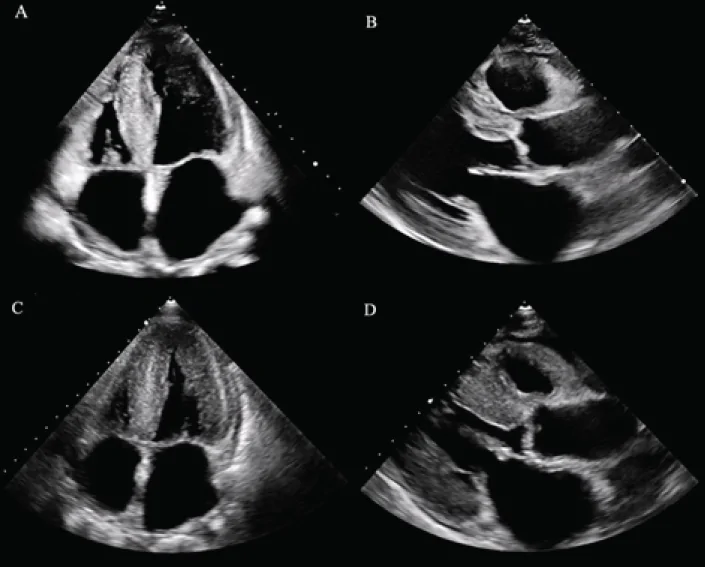
TTE demonstrating cardiac involvement in AL amyloidosis. TTE in both (A) apical four-chamber and (B) parasternal long-axis windows showing an increase in both left and right ventricular wall thickness. Repeat TTE after 9 months in both (C) apical four-chamber and (D) parasternal long-axis windows demonstrating worsening ventricular wall thickness. TTE: transthoracic echocardiography.

**Figure 7:**
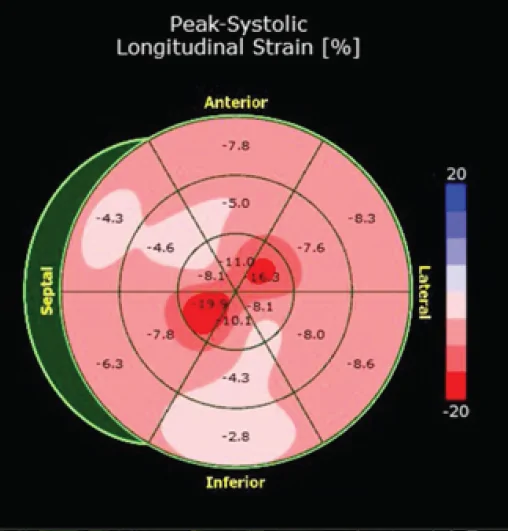
Global longitudinal strain analysis on transthoracic echocardiography demonstrating reduced overall value but with preservation of strain value at the apical region (i.e. a ‘cherry-on-top’ pattern), which commonly occurs in the context of cardiac amyloidosis.

**Table 1:** Serial investigations for diagnosis prior to chemotherapy.

Test	Month of disease progression				
	0	4	7	9	Reference range
**Complete blood count**					
Haemoglobin level (g/dL)	14.0	13.2	13.5	14.5	13–17
White blood cell count (10^9^/L)	7.4	7.4	5.8	5.9	4–10
Platelet count (10^9^/L)	317	318	356	267	150–410
**Liver function test**					
Total protein level (g/L)	60	48	53	54	64–83
Albumin level (g/L)	37	27	28	25	35–52
Alanine aminotransferase level (U/L)	35	53	48	39	<51
Alkaline phosphatase level (U/L)	99	99	101	132	40–129
**Renal function test**					
Urea level (mmol/L)	4.8	7.4	6.9	8.0	2.7-8.0
Creatinine level (μmol/L)	90	101	98	108	62-106
eGFR (mL/min/1.73 m^2^)	75	65	67	63	CKD-EPI ≥90: Normal or high 60-89: Mildly decreased 45-59: Mildly to moderately decreased 30-44: Moderately to severely decreased 15-29: Severely decreased <15: Kidney failure
**Coagulation profile**					
PT (s)	14.7	-	11.6	13.4	12–14
INR	1.1	-	1.0	1.0	<1.2
**Inflammatory markers**					
C-reactive protein level (mg/L)		-	<0.6	3.1	<5
ESR (mm/h)		-	48	-	0-15
**Urine test**					
UFEME protein level (g/L)	1.5	5.0	5.0	-	<0.3
RBC count (cell/μL)	27	4	62	-	<5
UPCR (g/mmol)	0.21	-	-	0.52	<0.05
**Cardiac markers**					
NT-proBNP level (pg/mL)	826	-	6373	8214	<125
hs-TnT level (ng/L)		-	145	-	<15
**Immunology (serum)**					
Kappa free light chain level (mg/L)		-	22.5	-	6.7-22.4
Lambda free light chain level (mg/L)		-	294	-	8.3-27.0
Kappa/lambda ratio		-	0.08	-	0.31-1.56
**Autoimmune screening**					
Anti-nuclear antibody		-	Neg	-	
Serum complement 3 level (mg/dL)		-	137	-	82-185
Serum complement 4 level (mg/dL)		-	58	-	15-53
Anti-myeloperoxidase level (p-ANCA)		-	0.2	-	<3.5
Anti-PR3 level (c-ANCA)		-	0.6	-	<2.0
**Cancer markers**					
Alpha-1-fetoprotein level (ng/mL)		-	5.1	-	≤9
Cancer AG19-9 level (U/mL)		-	8.5	-	≤35
Carcinoembryonic AG level (ng/mL)		-	1.9	-	≤3
Prostate-specific antigen level (ng/mL)		-	0.660	-	≤4
**Echocardiography**					
Ejection fraction^*^ (%)	-	56	-	46	>60
GLS (%)	-	-18.2	-	-8.7	<-18
LVH					
IVSd (cm)		1.4		1.7	0.6-1.2
LVPWd (cm)		1.2		1.9	0.6-1.2
Relative wall thickness		0.51		0.86	0.32-0.42
RVH^†^ (cm)		-		0.8	0.3-0.5

CKD-EPI: Chronic Kidney Disease Epidemiology Collaboration 2021, EGFR: estimated glomerular filtration rate, ESR: erythrocyte sedimentation rate, GLS: global longitudinal strain, hs-TnT: high-sensitivity troponin T, INR international normalised ratio, IVSd: interventricular septal thickness, LVH: left ventricular hypertrophy, LVPWd: left ventricular posterior wall thickness, NT-proBNP: N-terminal pro-B-type natriuretic peptide, PT: prothrombin time, RVH: right ventricular hypertrophy, UFEME: urine full examination and microscopic examination, UPCR: urine protein—creatinine ratio.

* Biplane Simpson’s method

† Right ventricular free wall thickness.

**Table 2:** Disease timeline, key clinical findings and diagnostic clues leading to the diagnosis of AL amyloidosis.

Organ system/domain	Key findings	Diagnostic clues/red flags
**Phase 1 – Month 0: Initial presentation**		
Cardiac	History of coronary artery disease Elevated NT-proBNP level: 826 pg/mL Cardiomegaly on chest radiography **Working diagnosis: Congestive cardiac failure**	The findings were attributed to known coronary artery disease and hypertensive heart disease; amyloidosis was not initially suspected.
Renal	Frank haematuria Proteinuria: 1.5 g/L eGFR 75 mL/min/1.73 m^2^ Renal ultrasound: No structural abnormality	Proteinuria as a systemic sign was initially overlooked.
Constitutional	No constitutional symptoms reported (no fever, weight loss, poor appetite or fatigue)	The absence of constitutional red flags directed the initial focus towards common conditions such as CCF
**Phase 2 – Month 4: Early organ involvement**		
Cardiac	ECHO: Concentric LVH, grade 2 diastolic dysfunction, preserved EF of 56%, borderline GLS of -18.2% **Working diagnosis: HFpEF**	HFpEF with unexplained LVH was a red flag for infiltrative cardiomyopathy.
Renal	Worsening eGFR: 65 mL/min/1.73 m^2^ Heavy proteinuria: 3.03 g/24 h Hypoalbuminaemia: 32 g/L **Working diagnosis: Nephrotic syndrome**	The combination of HFpEF and nephrotic syndrome was a red flag for AL amyloidosis.
**Phase 3 – Month 7: Overt disease progression**		
Soft tissue	Periorbital purpura Macroglossia	Pathognomonic signs that typically appear late in AL amyloidosis
Cardiac (advanced)	Worsened NT-proBNP level: 6373 pg/mL Elevated hs-TnT level: 145 ng/L ECG: Low-voltage QRS, pseudo-infarct pattern ECHO: Biventricular hypertrophy, reduced EF of 46%, severely impaired GLS of -8.7%, apical-sparing ‘cherry-on-top’ pattern	ECG-ECHO discordance and apical sparing on GLS were hallmarks of cardiac amyloid infiltration.
Renal	Persistent nephrotic-range proteinuria despite optimised ARB therapy and initiation of an SGLT2 inhibitor Stable eGFR: 62 mL/min/1.73 m^2^	Suggested an underlying infiltrative renal process, raising suspicion for renal AL amyloidosis.
**Phase 4 – Months 7–9: Targeted diagnostic workup**		
SPEP and immunofixation	Lambda light-chain paraprotein band (α2-β zone) Quantitation: <1 g/L No immune paresis Hypoalbuminaemia present	Lambda paraproteinaemia was consistent with an underlying plasma cell dyscrasia.
UPEP and immunofixation	Lambda light-chain paraprotein band detected in urine	Concordant with SPEP findings
Autoimmune, inflammatory and malignancy screening	ANA: Negative p-ANCA and c-ANCA levels: Normal Serum C3 level: Normal Serum C4 level: Mildly elevated^*^ AFP, CA 19-9, CEA and PSA levels: Normal	Excluded autoimmune, chronic inflammatory and malignancy- associated causes
Renal biopsy histopathology and immunofluorescence	Acellular amorphous deposits with nodular mesangial expansion Congo red positive with apple-green birefringence under polarised light Diffuse granular IgG, IgA, IgM, C3 and lambda deposition in the mesangium, capillary loops and arteriolar walls with lambda light-chain restriction; negative for C1q and kappa	Congo red-positive deposits confirmed amyloidosis; lambda restriction supported AL (lambda) subtype.
Renal biopsy immunohistochemistry	SAA-positive staining in the interstitium, vessel walls and perinephric adipose tissue	Although the SAA-positive stain suggested possible AA amyloidosis, there was no evidence of chronic inflammatory disease or infection associated with AA amyloidosis.
Cardiac scintigraphy (Tc-99m PYP scan)	No focal myocardial tracer uptake Visual grading score: Grade 0 No cardiac uptake, normal rib uptake	Negative scan excluded ATTR amyloidosis.
Bone marrow aspirate examination	The bone marrow sample showed normal cellularity with normal production of red blood cells, white blood cells and platelets. Plasma cells accounted for 2% of the total nucleated cells.	Low plasma cell burden; not supportive of other monoclonal disorder (i.e. multiple myeloma)
Bone marrow immunophenotyping	Clonal CD138+/CD38+ plasma cells accounted for 1.3% of the total nucleated cells. Approximately 78% showed an aberrant phenotype (CD45dim/CD38+/CD138+/CD56dim/CD19-). Lambda light-chain restriction was present (lambda+/kappa-).	Clonal lambda-restricted plasma cells confirmed an abnormal plasma cell clone, supporting the diagnosis of AL amyloidosis.
Bone marrow karyotyping/cytogenetics	Karyotype: 45,X [12]/46,XY [8] Mosaic loss of Y chromosome without additional abnormalities Age-related mosaic loss of Y chromosome; not clinically significant	Advanced age male karyotyping. Excluded mutant/hereditary aetiology
**Phase 5 – Month 9: Final diagnosis and staging**		
Multiorgan (cardiac + renal)	The integrated clinical, laboratory and biopsy findings for amyloid typing assessment were consistent with AL amyloidosis. **Cardiac staging: European-modified Mayo 2004 stage Ilia** (hs-TnT level: 145 ng/L, NT-proBNP level: 6373 pg/ mL)	**Final diagnosis: Systemic AL (lambda) amyloidosis with cardiac and renal involvement**
**Phase 6 – Months 9–12: Follow-up and outcome**		
Haematology review: Worsening kidney function and persistent heart failure symptoms, suggesting a suboptimal treatment response. The patient was referred to cardiology for optimisation of heart failure management and diuresis. Cardiology review: The patient was admitted for inpatient diuresis due to decompensated heart failure. The clinical course was complicated by respiratory distress and multiple-organ dysfunction. **Final outcome:** Death from sepsis with multiple-organ dysfunction secondary to pneumonia.		

AFP: alpha-1-fetoprotein, AL: amyloid light-chain, ANA: anti-nuclear antibody, ANCA: antineutrophil cytoplasmic antibody, ARB: angiotensin receptor blocker, ATTR: transthyretin, CA 19-9: cancer AG19-9, CEA: carcinoembryonic AG, CCF: congestive cardiac failure, ECG: electrocardiogram, ECHO: echocardiogram, EF: ejection fraction, eGFR: estimated glomerular filtration rate, GLS: global longitudinal strain, HFpEF: heart failure with preserved ejection fraction, hs-TnT: high-sensitivity troponin T, IHC: immunohistochemistry, LVH: left ventricular hypertrophy, NT-proBNP: N-terminal pro-B-type natriuretic peptide, PSA: prostate- specific antigen, PYP: pyrophosphate, SAA: serum amyloid A, SPEP: serum protein electrophoresis, UPEP: urine protein electrophoresis.

* A mildly elevated C4 level is a non-specific finding and was not consistent with active inflammatory disease in this clinical context.

**Table 3:** Diagnostic evidence for AL amyloidosis in accordance with the ICC 2022 criteria.

No.	Diagnostic criteria	ICC 2022 requirement	Patient findings	Interpretation
1.	Clinical suspicion	Systemic involvement related to amyloid	Nephrotic syndrome Heart failure Periorbital purpura Macroglossia	Strongly supportive
2.	Tissue confirmation	Congo red staining showing apple-green birefringence in any tissue	Renal biopsy: Congo red positive	Confirmed amyloid deposits
3.	Evidence of plasma cell clone proliferative disorder	Monoclonal light chain on immunofixation, abnormal FLC ratio or clonal plasma cells on bone marrow	FLC: Monoclonal lambda Positive immunofixation Bone marrow: Clonal plasma cell population	Confirmed plasma cell clone
4.	Amyloid typmg	Direct examination of the amyloid through immunohistochemistry or mass spectrometry confirming light-chain type	Immunohistochemistry: The AA pattern was inconsistent with clinical context, and mass spectrometry was unavailable in Malaysia. Immunofixation and bone marrow: Confirmed lambda light-chain restriction	Supported AL type
5.	Exclusion of other amyloid subtypes^*^	Exclude ATTR, hereditary or AA amyloidosis	Negative Tc-99m PYP No clinical/laboratory evidence of chronic inflammation	Confirmed AL subtype
6.	Multiorgan involvement and severity^*^	Objective evidence of organ dysfunction from amyloid infiltration (cardiac, renal, hepatic, neurologic or gastrointestinal)	Elevated cardiac biomarkers Renal impairment with nephrotic-range proteinuria	Significant multiorgan involvement
7.	Final diagnostic classification	Criteria 1–4 met with supportive exclusion of non-AL forms	All ICC 2022 criteria fulfilled for systemic AL amyloidosis	Definite diagnosis

AA: amyloid A, AL: amyloid light chain, ATTR: transthyretin, FLC: free light chain, ICC: International Consensus Classification, Tc-99m PYP: technetium-99m pyrophosphate.

* Supportive investigations but not mandatory for the ICC 2022 diagnostic criteria.

### Therapeutic intervention

Following the diagnosis, approximately 9 months after symptom onset, the haematology team reviewed the patient’s condition and started anti-clonal chemotherapy with cyclophosphamide, bortezomib and dexamethasone (CyBorD). The patient and his family were counselled on the rationale for bortezomib-based therapy, the anticipated need for multiple cycles and potential adverse effects. Daratumumab-based therapy was also discussed as a treatment option, but the selection was influenced by local accessibility and financial considerations. After counselling, the patient and his family opted to proceed with CyBorD.

### Follow-up and outcomes

Three months after treatment initiation, haematology review during the third cycle of CyBorD demonstrated worsening renal function and persistent congestive heart failure symptoms, suggesting a suboptimal response to therapy. Given the poor haematological and clinical responses, a funding application for daratumumab (approximately RM 17,000 per injection) was submitted, and the patient was concurrently referred to cardiology for optimisation of heart failure management and diuresis. At subsequent outpatient cardiology review, decompensated heart failure was identified, prompting admission for decongestion.

Despite these interventions, his condition deteriorated with worsening respiratory distress and multiple-organ dysfunction, and he was thus unfit to continue with the scheduled fourth cycle of CyBorD. He subsequently passed away from sepsis with multiple-organ dysfunction secondary to bacterial pneumonia.

## Discussion

This case demonstrates how a rare systemic disease can initially masquerade as common primary care presentations, resulting in progressive diagnostic complexity and delayed recognition. Its complex diagnostic trajectory highlights three critical insights for family physicians: Recognise clinical discordance and pathognomonic features; understand how delayed diagnosis worsens prognosis; and appreciate the coordinating role of family physicians in managing rare systemic disease.

AL amyloidosis, known as the ‘great masquerader’, deposits amyloid fibrils in the cardiac interstitium, renal mesangium, hepatic sinusoids, bone marrow perivascular spaces and skeletal muscle connective tissues, replacing normal architecture and causing progressive dysfunction.^7^ It often presents with routine complaints such as oedema and haematuria yet rapidly evolves into multisystem failure once cardiac and renal involvements accelerate. Early signs lack specificity, which complicates diagnosis. Limited epidemiological data on the Asian population further limit accurate incidence assessment, although regional studies demonstrate multiorgan involvement in most cases.^8^

Two patterns of clinical discordance were pivotal. First, concentric LVH on ECHO with low-voltage QRS complexes on ECG strongly suggests infiltrative cardiomyopathy rather than hypertensive heart disease.^4^ Second, the concurrent presentation of HFpEF and non-diabetic nephrotic-range proteinuria in a patient with previously controlled hypertension represents an atypical disease trajectory. These discordant findings, combined with intolerance to standard heart failure therapy and worsening cardiac biomarkers, substantially narrowed the differential diagnosis.

Intolerance to standard heart failure therapy is a recognised clinical red flag for cardiac amyloidosis. Due to restrictive physiology and fixed stroke volume, patients depend on heart rate and higher filling pressures to maintain cardiac output; consequently, beta-blockers and vasodilators may precipitate hypotension, fatigue and low-output symptoms. Autonomic dysfunction further contributes to orthostatic symptoms and poor drug tolerance.^9^ The patient’s giddiness after beta-blocker initiation despite normal blood pressure and pulse illustrates this under-recognised clinical clue. ECG features include low-voltage QRS in limb leads and Q waves in precordial leads mimicking prior myocardial infarction, although sensitivity varies from 28% to 76%.^5^ Combined with other systemic features and the rare combination of macroglossia and periorbital purpura,^2^ this case offers a textbook cluster of diagnostic clues seldom encountered in real-world family practice.

After tissue confirmation of amyloid deposition, accurate amyloid typing is essential because treatment and prognosis differ markedly. In Malaysia, this is challenging because mass spectrometry, the gold standard method, is not locally available; therefore, practical alternatives include a combination of IHC, serum and urine immunofixation and bone marrow examination, together with systematic exclusion of AA and ATTR amyloidoses.^10^ IHC-based typing must be interpreted cautiously, as antibody cross reactivity, limited specificity and fixation related epitope masking can yield misleading or inconclusive results.^11,12^ AA amyloidosis typically occurs in the setting of chronic inflammatory or infectious disease, whereas AL amyloidosis is associated with an underlying monoclonal plasma cell disorder and more often involves lambda light chains; bone marrow examination is thus important to assess plasma cell burden and exclude other monoclonal disorders.^13^ Key distinctions between AL and AA amyloidoses are summarised in Table 4.^2,14^ Confirmation of AL amyloidosis follows the ICC framework, which requires the following: (1) systemic organ involvement attributable to amyloid; (2) Congo red-positive tissue biopsy; (3) evidence that the amyloid is light chain-related on IHC or mass spectrometry; and (4) evidence of a clonal plasma cell disorder.^15^ A complementary algorithm by Gertz et al. recommends a stepwise approach: Identify unexplained infiltrative cardiomyopathy or HFpEF, proteinuria, neuropathy, hepatomegaly or plasma cell dyscrasia; screen for monoclonal protein using serum and urine immunofixation with serum free light-chain assay; and, if monoclonal gammopathy is present, confirm amyloid via Congo red-positive biopsy, establish AL subtype via IHC or mass spectrometry and proceed to disease staging.^2^

**Table 4:** Distinguishing features of AL versus AA amyloidosis.

Feature	Points towards AL amyloidosis	Points towards AA amyloidosis
Underlying disease	Plasma cell dyscrasia	Chronic inflammatory or infectious disease (e.g. TB, RA, IBD or malignancy)
Monoclonal protein screening	Positive	Negative
CRP level/ESR	Normal or mildly elevated	Persistently and markedly elevated
Bone marrow plasma cells	Clonal plasma cells with kappa or lambda restriction	No clonal cells; reactive plasmacytosis only
Dominant organ involvement	Heart > kidneys > peripheral nerves > GI	Kidneys > spleen/liver > GI; cardiac involvement is rare.
Cardiac involvement	Common and often an early feature	Uncommon, usually late
Macroglossia/periorbital purpura	Pathognomonic of AL amyloidosis	Typically absent
Age at presentation	Any adult; median age of60-65 years	Often younger with long-standing inflammatory disease
Family history of inflammatory disease	Usually negative	May be positive, particularly JIA or IBD

AA: amyloid A, AL: amyloid light chain, CRP: C-reactive protein, ESR: erythrocyte sedimentation rate, GI: gastrointestinal, IBD: inflammatory bowel disease, JIA: juvenile idiopathic arthritis, RA: rheumatoid arthritis, TB: tuberculosis.

Diagnostic delays are common and associated with poorer prognosis, particularly with cardiac involvement. Only 26% of patients are diagnosed within 1 year of symptom onset.^2^ Family physicians serve as the first points of contact for 64.9% of patients, yet only 18.1% and 24.5% receive timely nephrology and cardiology referrals, respectively.^16^ Reported contemporary data suggest that the median time from symptom onset to diagnosis of AL amyloidosis remains approximately 6 months and that neuropathic or musculoskeletal presentations are associated with the longest delays of up to 156 months.^17^ In this case, the patient’s 9-month interval to diagnosis underscores the persisting challenge of early recognition. The American Society of Hematology (ASH) 2026 diagnostic guideline outlines structured recommendations to enhance clinical suspicion.^18^ However, in Malaysia, access to advanced amyloid typing techniques such as mass spectrometry remains limited in many resource-constrained settings, creating additional diagnostic challenges. In response to this gap, we propose a practical diagnostic flowchart to help family physicians recognise red flags (as outlined by the ASH 2026), initiate baseline assessment and define a clear referral pathway for suspected AL amyloidosis in primary care (Figure 8). Early identification of warning signs in primary care can meaningfully shorten the time to diagnosis. The progressive nature of the findings required iterative clinical reassessment, a strength of longitudinal primary care.

**Figure 8:**
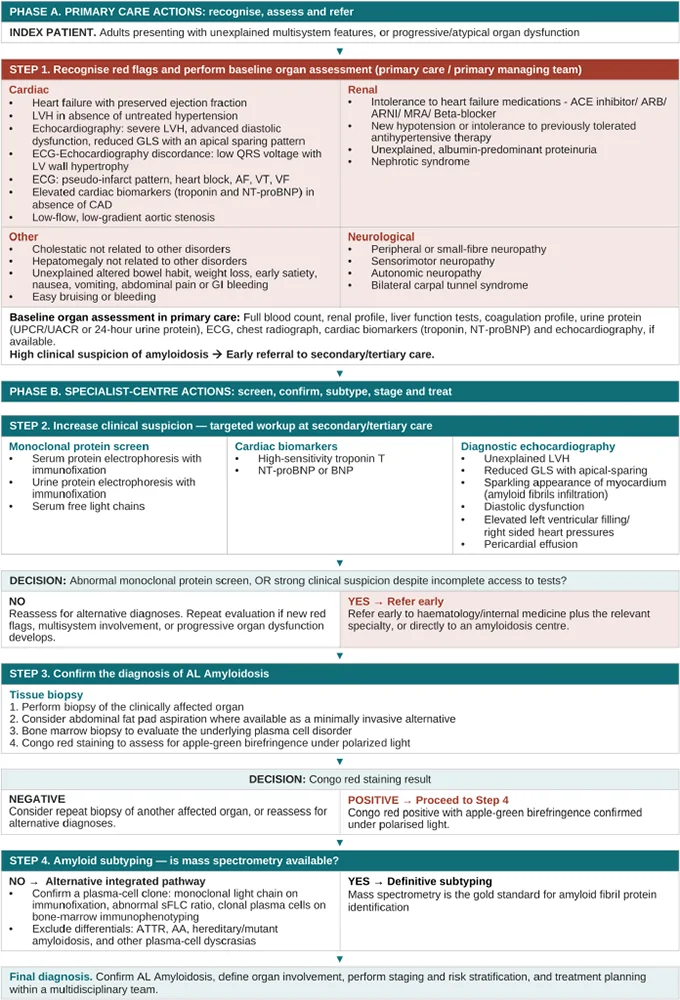
Diagnostic flowchart for suspected AL amyloidosis in primary care. AA: amyloid A, ACE: angiotensin-converting enzyme, ALP: alkaline phosphatase, ARB: angiotensin receptor blocker, ARNI: angiotensin receptor neprilysin inhibitor, ATTR: transthyretin, BNP: B-type natriuretic peptide, CMR: cardiac magnetic resonance, ECG: electrocardiogram, FBC: full blood count, GI: gastrointestinal, GLS: global longitudinal strain, hs-TnT: high-sensitivity troponin T, IHC: immunohistochemistry, LFT: liver function test, LV: left ventricle, LVH: left ventricular hypertrophy, NT-proBNP: N-terminal pro-B-type natriuretic peptide, MRA: mineralocorticoid receptor antagonist, PYP: pyrophosphate, sFLC: serum free light chain, SIFE/UIFE: serum/urine immunofixation electrophoresis, SPEP/UPEP: serum/urine protein electrophoresis, SPECT: single-photon emission computed tomography, UACR: urine albumin—creatinine ratio, UPCR: urine protein—creatinine ratio. This figure is intended to support early recognition and referral from primary care; it does not replace specialist diagnostic confirmation or local referral protocol. The red-flag signs and criteria for raising clinical suspicion were based on the recommendation from the American Society of Hematology 2026 guideline on the diagnosis of AL amyloidosis.

The prognosis of AL amyloidosis is largely driven by the extent of cardiac involvement. The European-modified Mayo 2004 staging system stratifies patients according to cardiac biomarker burden and predicts early mortality risk more accurately; in this case, the hs-TnT level of 145 ng/L and NT-proBNP level of6373 pg/mL correspond to stage IIIa disease, with an estimated median survival of about 24 months.^19^ More recently, the AL International Staging System (AL-ISS) has been introduced in the daratumumab era,^20^ and both systems demonstrate substantially reduced survival with advancing cardiac stage, down to around 4 months in European-modified Mayo stage IIIb and 7 months in AL-ISS stage IIIc (Table 5), underscoring the poor prognosis of advanced disease and the critical need for early diagnosis.

**Table 5:** Staging and prognosis of AL amyloidosis.

Stage	European-modified Mayo 2004	Median survival (month)	AL-ISS	Median survival (month)
I	NT-proBNP and hs-TnT levels below the threshold	130	NT-proBNP and hs-TnT levels below the threshold	NR
II	NT-proBNP level of ≥332 ng/L **or** hs-TnT level of ≥50 ng/L	54	NT-proBNP level of ≥332 ng/L **or** hs-TnT level of ≥50 ng/L	NR
IIIa	NT-proBNP level of 332-8500 ng/L **and** hs-TnT level of ≥50 ng/L	24	NT-proBNP level of 332-8500 ng/L **and** hs-TnT level of ≥50 ng/L	67
IIIb	NT-proBNP level of ≥8500 ng/L **and** hs-TnT level of ≥50 ng/L	4	NT-proBNP level of ≥8500 ng/L **and** hs-TnT level of ≥50 ng/L and GLS of <-9%	26
IIIc	-	-	NT-proBNP level of ≥8500 ng/L **and** hs-TnT level of ≥50 ng/L **and** GLS of ≥-9%	7
Patient results: hs-TnT level: 145 ng/L; NT-proBNP level: 6373 pg/mL^*^; GLS: –8.7%				
Correspond to **stage IIIa**				

GLS: global longitudinal strain, hs-TnT: high-sensitivity troponin T, NT-proBNP: N-terminal pro-B-type natriuretic peptide, NR: not reported.

* NT-proBNP units: pg/mL and ng/L are equivalent.

The treatment strategies for AL amyloidosis include systemic chemotherapy and autologous stem cell transplantation. The current first-line therapy in many centres is daratumumab combined with CyBorD, which provides better haematological responses and survival than CyBorD alone.^21^ However, regimen selection remains individualised and depends on disease stage, drug availability, cost and patient fitness. In this case, CyBorD was selected after considering local availability and financial constraints. Shared decision-making with the patient and his family was important to ensure that the treatment aligned with their preferences and resources. Despite the initiation of CyBorD, the patient died from infectious complications, a common terminal event in advanced AL amyloidosis due to combined disease- and treatment-related immunocompromise.^22^ Early recognition is therefore critical, as timely therapy can suppress the amyloidogenic plasma cell clone, limit further amyloid deposition and organ damage and improve clinical outcomes.

### Take-home message

Family physicians should suspect AL amyloidosis in patients presenting with unexplained HFpEF, nephrotic-range proteinuria, ECG-ECHO discordance, poor tolerance to standard heart failure therapy or characteristic findings such as periorbital purpura and macroglossia. Early recognition and referral may substantially improve treatment opportunities and survival.

### Patient perspective

The following perspective was provided by the patient’s sons and edited only for grammar and clarity:

‘At first, we thought his symptoms were related to common heart problems, as he had leg swelling, tiredness and, later, worsening breathlessness. The symptoms developed gradually, and it was difficult for us to understand that they could be signs of a rare and serious illness. He attended several clinic appointments and underwent multiple investigations before the diagnosis was established. The process was challenging for our family. We hope that sharing his experience will help doctors to detect this disease easier and help patients receive the best medical care possible’.

## Conclusion

This case highlights both the diagnostic challenges of AL amyloidosis and the opportunity for earlier recognition in primary care. As healthcare gatekeepers, family physicians require heightened awareness of AL amyloidosis, streamlined diagnostic pathways and institutional support to improve outcomes. Implementing concise primary care checklists for amyloidosis suspicion, clear referral pathways to specialised centres and routine integration of prognosis discussions with advance care planning may enhance diagnostic timeliness and patient outcomes.

## Clinical pearls

- ECG-ECHO discordance indicates infiltrative cardiomyopathy until proven otherwise.
- HFpEF plus nephrotic syndrome should prompt screening for amyloidosis.
- Periorbital purpura and macroglossia are pathognomonic but late signs.
- Poor beta-blocker tolerance in presumed HFpEF is a red flag for restrictive physiology.

## References

[1] Buxbaum JN, Eisenberg DS, Fändrich M (2024). Amyloid nomenclature 2024: update, novel proteins, and recommendations by the International Society of Amyloidosis (ISA) Nomenclature Committee. Amyloid.

[2] Gertz MA, Dispenzieri A (2020). Systemic amyloidosis recognition, prognosis, and therapy: a systematic review. JAMA.

[3] Sanchorawala V (2024). Systemic light chain amyloidosis. N Engl J Med.

[4] Roslan A, Kamsani SH, Nay TW (2018). Echocardiographic and electrocardiographic presentations of patients with endomyocardial biopsy-proven cardiac amyloidosis. Med J Malaysia.

[5] Zhang KW, Vallabhaneni S, Alvarez-Cardona JA, Krone RJ, Mitchell JD, Lenihan DJ (2021). Cardiac amyloidosis for the primary care provider: a practical review to promote earlier recognition of disease. Am J Med.

[6] Szor RS, Castelli JB, Schuch RA, Carvalho VM, Rocha V (2025). Assessing the consistency of mass spectrometry, a clinical-laboratory model, and immunohistochemistry in amyloid subtyping: a Brazilian experience. Clin Proteomics.

[7] Kim MJ, Baek D, Truong L, Ro JY, Kurouski D (2019). Pathologic findings of amyloidosis: recent advances. Amyloid Diseases.

[8] Yoon SE, Kim D, Choi JO (2023). A comprehensive overview of AL amyloidosis disease characteristics accumulated over two decades at a single referral center in Korea. Int J Hematol.

[9] Hwa YL, Fogaren T, Sams A (2019). Immunoglobulin light-chain amyloidosis: clinical presentations and diagnostic approach. J Adv Pract Oncol.

[10] Alias AM, Shariff RER, Saidon N (2024). Cardiac amyloidosis: everywhere, but nowhere. J Asian Pac Soc Cardiol.

[11] Colombat M, Gaspard M, Camus M (2022). Mass spectrometry-based proteomics in clinical practice amyloid typing: state-of-the-art from a French nationwide cohort. Haematologica.

[12] Riefolo M, Conti M, Longhi S, Fabbrizio B, Leone O (2022). Amyloidosis: what does pathology offer? The evolving field of tissue biopsy. Front Cardiovasc Med.

[13] Fend F, Dogan A, Cook JR (2023). Plasma cell neoplasms and related entities—evolution in diagnosis and classification. Virchows Arch.

[14] Mirioglu S, Uludag O, Hurdogan O (2024). AA amyloidosis: a contemporary view. Curr Rheumatol Rep.

[15] Leung N, Nasr SH, Sethi S (2012). How I treat amyloidosis: the importance of accurate diagnosis and amyloid typing. Blood.

[16] Lousada I, Comenzo RL, Landau H, Guthrie S, Merlini G (2015). Light chain amyloidosis: patient experience survey from the Amyloidosis Research Consortium. Adv Ther..

[17] Bradbury H (2025). COMy 2025: diagnosis delays in amyloid light-chain amyloidosis. European Medical Journal..

[18] Kukreti V, Seftel M, Aguirre MA (2026). American Society of Hematology 2026 guidelines on diagnosis of light chain amyloidosis. Blood Adv..

[19] Charlinski G, Steinhardt M, Rasche L (2024). Outcomes of modified Mayo stage IIIa and IIIb cardiac light-chain amyloidosis: real-world experience in clinical characteristics and treatment—67-patient multicenter analysis. Cancers (Basel)..

[20] Khwaja J, Kirkwood AA, Milani P (2026). New validated staging system for light chain (AL) amyloidosis with stage IIIC defining ultra-poor risk: AL International Staging System. J Clin Oncol..

[21] Kastritis E, Palladini G, Minnema MC (2026). Daratumumab-bortezomib-cyclophosphamide-dexamethasone in newly diagnosed amyloidosis: ANDROMEDA final survival analysis. Blood..

[22] Jaccard A, Comenzo RL, Hari P (2014). Efficacy of bortezomib, cyclophosphamide and dexamethasone in treatment-naive patients with high-risk cardiac AL amyloidosis (Mayo Clinic stage III). Haematologica..

